# Impact of racial discrimination in education and other adverse childhood experiences on black students’ mental health and wellbeing: an interpretative phenomenological analysis study

**DOI:** 10.1080/17482631.2025.2507754

**Published:** 2025-05-23

**Authors:** Nkasi Stoll, Sunehna Kayn, Heidi Lempp, Stephani Hatch

**Affiliations:** a Psychological Medicine, Institute of Psychiatry Psychology and Neuroscience, King’s College London, London, UK; b Faculty of Education and Society, Culture, Communication and Media, University College London, London, UK; c Inflammation Biology, Faculty of Life Sciences & Medicine, King’s College London, London, UK; d Economic and Social Research Council Centre for Society and Mental Health, King’s College London, London, UK

**Keywords:** Student mental health, Black students, narrative interviews, interpretative phenomenological analysis, race-based stress, adverse childhood experiences

## Abstract

**Introduction:**

Black school students in the United Kingdom (UK) are exposed to racialized personal, institutional and systemic factors (e.g. racism and misogyny) that may impact their mental wellbeing and educational experiences. Minimal research exists to understand how racism and other adverse childhood experiences interact to shape students’ mental health and wellbeing as they progress through education before commencing university studies, which this study aimed to achieve.

**Methods:**

Biographical Narrative Interpretive Method (BNIM) interviews (*n* = 15) were completed with Black UK university students who self-reported having struggled with mental health at school. Data were analysed using Interpretative Phenomenological Analysis (IPA).

**Results:**

Three main themes were derived from Interpretative phenomenological analysis (IPA), pertaining to the students’ experiences of adverse childhood experiences, racism-related stressors in education, and coping strategies.

**Discussion:**

The findings add value by offering recommendations for psychologists, educators, and policymakers to address racism and poor mental wellbeing in schools and to improve experiences and outcomes for Black students. Recommendations include self-reflection tools, funding for mental wellbeing interventions and resources, and enhancing professional courses to incorporate anti-racist curricula and practices.

## 1. Introduction

Mental health shapes students’ learning, work and social experiences, and their ability to cope with stressful life experiences (World Health Organization, 2022). The transition from school to university often exposes students to stressors that can trigger health risk behaviours (e.g., alcohol misuse, risky sexual practices) and poor mental health (e.g., psychological distress, mood disorders). Students may be at a particular risk due to unpreparedness, separation from family, fear of the unknown, poor social support, and inaccessible wellbeing services (Cage et al., 2021; Chung & Hudziak, 2017; Mofatteh, 2021). Students come from diverse backgrounds with varying privileges and barriers that impact their educational experiences and outcomes (Bernard et al., 2022). Additionally, Black students face institutional racism and racial discrimination, which may impact their mental health and access to mental health and educational support services (Arday, 2018; Sancho & Larkin, 2020; Stoll, Jieman, et al., 2023; Stoll, Yalipende, Byrom, et al., 2022).

In the United Kingdom (UK), critical race theorists have asserted that education policies are designed and implemented to disadvantage Black students by failing to address institutional racism, ignoring cultural differences, and maintaining white privileges (Gillborn et al., 2017; Warmington, 2020). For example, many UK schools punish students for wearing their hair in natural afro styles, braids, cornrows and plaits by implementing “racially neutral” policies and practices that fail to consider race, ethnicity and culture (O’Neill et al., 2025). These “racially blind” policies work to maintain the racial achievement gap between white advantaged and disadvantaged racially minoritized students (Harven, 2018). National UK data have exposed that Black students are more likely to be excluded or suspended from school, and educated in pupil referral units,^1^ compared to white British or Asian students (GOV.UK, 2024a, 2024b), possibly due to educators’ negative racial stereotypes (Graham et al., 2019). A UK survey found that 95% of young Black people had heard or witnessed racist language at school, with the main barrier to success being teachers’ negative perceptions (50%) and racism from their peers and staff (49%) (YMCA, 2020). According to studies in the United States of America (US), persistent exposure to racism has been linked to increased symptoms of depression and anxiety (Smith, Hung, et al., 2011; Smith, Yosso, et al., 2011; Smith et al., 2020). While confronting racist remarks can reduce negative self-esteem and anxiety (Williams, 2018), this does not diminish the impact of institutional racism on Black students (Boone Blanchard et al., 2021).

To examine the impact of stress on Black students, the authors used the Culturally-Informed Adverse Childhood Experiences Framework (C-ACE) (Bernard et al., 2020). The Adverse Childhood Experiences (ACE) model suggests that childhood exposure to potentially traumatic events (e.g., historical trauma, witnessing violence) can have a dose-dependent impact on health behaviours and health, neurodevelopment, and social, emotional and cognitive functioning, heightening the risk of mental and physical illness (Centers for Disease Control and Prevention, 2021; Hughes et al., 2017). ACEs are consistently linked to adult mental health disorders, particularly in individuals with multiple types of adverse experiences (Hatch, 2005; Sahle et al., 2022). Black students who report ACEs are more likely to recall racial discrimination during their lifetime (Dorvil et al., 2021; Helminen et al., 2022; Lemon et al., 2022; Marks et al., 2021), potentially increasing their risk of depression and/or anxiety, symptoms compared to their non-racially minoritised peers (Dorvil et al., 2021; Helminen et al., 2022; Lemon et al., 2022; Marks et al., 2021). The C-ACE framework posits that historical racism (e.g., inter-generational racism-related stress) continues to shape current sociocultural conditions (e.g., poverty) and biopsychological vulnerability (e.g., dispositional risk factors), thereby increasing the risk of ACEs (e.g., racial discrimination) (Bernard et al., 2020). In an educational context, these experiences expose Black students to racism-related stressors (e.g., racial discrimination in classrooms), worsen biopsychological vulnerability (e.g., cortisol sensitivity) and health risk behaviours (e.g., smoking), that in turn impair mental health outcomes and increase vulnerability to future ACEs in education and other social settings (e.g., poor educational attainment) (Bernard et al., 2020).

Much of the relevant literature, theories, and frameworks on the intersection between racism, mental health and education have focused on the US educational system, which may not apply to UK-specific contexts that differ in terms of access, equity, curriculum, and assessments. Notably, both the UK and the US curriculum are Eurocentric, with British colonialist and imperialist ideologies embedded in the architectural design and pedagogy in UK schools (Sengupta & King, 2024). Qualitative inquiry is vital to understand how racism-related ACEs at the interpersonal and institutional levels interact with other ACEs to uniquely and cumulatively impact mental health at key developmental stages to know how best to support and resource potentially vulnerable students transitioning into university. A lack of appropriate and timely mental health support can lead to the progression into other co-morbid disorders, substance use disorders, self-harm, suicidal ideation, and suicide attempts (Boden & Foulds, 2016; Eskin et al., 2016; Hawton et al., 2012; McGorry et al., 2011). Therefore, the current study aimed to explore the life events and experiences that shape Black students’ mental health and wellbeing before commencing UK university studies.

## 2. Methods

### 2.1. Study design

This article is part of a national qualitative study on the mental health and wellbeing experiences of Black UK undergraduate, master’s and doctoral university students (Stoll, Yalipende, Arday, et al., 2022). The current study is an adapted version of the Biographic Narrative Interpretive Method (BNIM) interviews (Wengraf, 2001, 2008) for data collection and Interpretative Phenomenological Analysis (IPA) design (Smith et al., 2022) for data analysis.

This design was chosen because the open narrative interview process of BNIM allowed students to begin, construct, and end their stories on their own terms (Wengraf, 2018). This produced rich, in-depth narrative data that are necessary for IPA analysis (Frechette et al., 2020). The follow-up questions (i.e., narrative/N-pointed questions) enabled the exploration of unique narratives that are particularly significant, nuanced, or unclear. This focused approach helped to refine the analysis to interpret not only what was said, but also how the students communicated their stories, and the meanings attached to those expressions.

### 2.2. Sampling criteria and recruitment

Purposive sampling (Smith & Osborn, 2003) was used to recruit 15 Black university students who met the inclusion criteria. The inclusion criteria were kept purposely broad to allow a wide range of Black students from different backgrounds to be eligible for study inclusion as the research progressed. Participants had to self-identify as Black (Caribbean, African, and mixed heritage with Black) and self-reported to have struggled with their mental health at some point during their UK education journey. They needed to be aged 18 or above and within a year of graduating from, or within a year of dropping out of a university course. Participants could be of any nationality.

Students were recruited via invitation emails to student unions, student groups, student services, and social media accounts. Potential participants were sent an Information Sheet and Consent Form and were invited to complete an Expression of Interest Form with sociodemographic information to determine their eligibility for the study. After the interview, participants were sent a £20 e-voucher and an e-card (Ellard-Gray et al., 2015).

### 2.3. Participants

Participants (*n* = 15) were current university students, and their ages ranged from 18 to 50 years old. All had attended secondary school in the UK and were currently enrolled at or had previously graduated from one of eleven UK universities. Table I summarizes the participants’ sociodemographic characteristics.

**Table I.** Participants characteristics.

Characteristics	n/15	%
**Race/ethnicity**		
Black African	6	40%
Black Caribbean	5	33%
Black Caribbean and African	2	13%
Black African and white European	1	7%
Black Caribbean and white European	1	7%
**Age range**		
18–24	9	60%
25–35	5	33%
36–50	1	7%
**Gender**		
Female	8	53%
Male	5	33%
Non-binary	2	13%
**Nationality**		
UK	10	67%
International/Dual national	5	33%
**Pre-university school location**		
England	7	47%
Northern Ireland	3	20%
Scotland	3	20%
Wales	2	13%
**Current level of study**		
Undergraduate	6	40%
Masters	4	27%
Doctorate	5	33%

### 2.4. Data collection

Interviews were conducted between December 2020 and June 2021 using an adapted two sub-session BNIM technique (Stoll, Yalipende, Byrom, et al., 2022). Interviews were conducted virtually using Microsoft Teams and recorded with the students’ permission. The interview lengths ranged from 1 h and 30 min to 2 h and 15 min. Recordings were transcribed by NS using Microsoft Teams’ automated speech-to-text transcription program, and a sample was reviewed and verified for accuracy by the study team.

The students were asked a single question called a “Single Question Inducing Narrative” (SQUIN) (Wengraf, 2001, 2008): “*Please can you tell me the story of your mental health experiences before university*”. NS demonstrated passive listening by keeping their camera on, turning their microphone off, and avoiding interruptions. As the student talked, NS took notes on “Particular Incident Narratives” (PINs) (Wengraf, 2001, 2008) capturing significant events related to mental health and wellbeing prior to commencing university studies using the participants’ own keywords (e.g., schoolteachers using racial slurs). During a 15-min break NS identified “narrative/N-pointed questions” (Wengraf, 2001, 2008) to clarify or pick up on PINs (e.g., “You mentioned a teacher in year 6 used the n-word, do you remember how that made you feel at the time?”). The second session began with NS asking the student to reflect on the identified PINs and continued with the N-pointed questions. NS demonstrated active listening by encouraging elaboration, asking for examples, and showing non-verbal responses (e.g., nodding).

A BNIM pilot study was conducted with two Black students to assess the comprehension and relevance of questions, timing and research burden (Malmqvist et al., 2019). No amendments were made to the study process, and the pilot data were included in the analysis.

### 2.5. Ethics, data protection and participant safety

Full ethical approval was obtained from King’s College London Psychiatry, Nursing and Midwifery Research Ethics Subcommittee (Rec Ref: 20489, Project Ref: MOD-20/21–20489, 24 June 2021). Written informed consent was obtained from all students to participate in the study and for their anonymous stories to be published. Pseudonyms were assigned to all universities and participants.

Three participant safeguarding strategies were planned for when participants became distressed depending on the level of distress, (i) offer a break, (ii) end the interview and re-schedule or (iii) contact emergency services. Six participants required multiple breaks, while the other two strategies were not required. A list of UK mental health and wellbeing services offering support for Black students was collated and sent to all students after the interviews.

### 2.6. Data analysis

The research team employed multiple strategies to ensure validity, rigour and credibility. NS (researcher and interviewer) created multiple questions to record and challenge their assumptions, beliefs, and attitudes about the design, methods, participants, and findings in a self-reflective log (Supplementary Material 1). An advisory team of five Black students and two teachers consulted on the research design, materials, analysis, and recommendations to ensure that all decisions and outputs were respectful and relevant (Durose et al., 2012). The authors reviewed samples of transcripts and codes, to provide credibility checks to the analysis (McMahon & Winch, 2018). Participants were sent copies of a summary of the analysis to review, six replied validating the themes (Birt et al., 2016).

**Figure 1.**
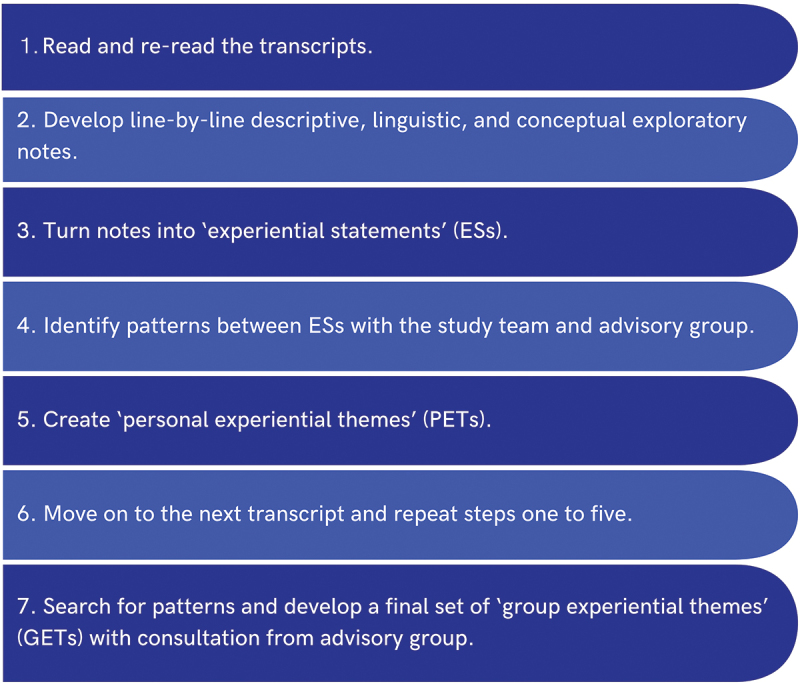
Summary of the interpretive phenomenological approach (Smith et al., 2022) steps used in the current study.

Data analysis was led by NS using IPA (Smith et al., 2022) (Figure 1). The first step of involved reading and re-reading the transcripts. NS then focused on developing line-by-line descriptive, linguistic and conceptual exploratory notes that remained close to the participants’ own words. The third step involved mapping the relationships, connections and patterns between exploratory notes to create “experiential statements” (ES). Experiential statements represented recurring patterns of meaning (i.e., ideas, thoughts and feelings) about the participants’ mental health experiences. Experiential statements were then grouped based on how much they seemed to relate to each other. The fifth step involved creating “personal experiential themes” (PETs), which were multiple experiential statements that captured patterns in participants’ emotional and cognitive experiences. This iterative process involved monthly online meetings between NS, SH, and HL during which they collaboratively discussed and decided which statements and themes closely reflected the participants’ narratives. Any disagreements or uncertainty were brought to the advisory team, who provided feedback on the emerging findings. Statements and themes that did not answer the research question, fit well with the emerging narrative, or had weak evidence base were discarded. Stages one to six were repeated for each transcript.

Finally, NS searched for patterns of convergence and divergence across the personal experiential themes to develop a set of main themes that represented the shared and unique participant mental health experiences. Verbatim extracts were selected and are provided throughout the results to ensure transparency and sensitivity to the raw data.

### 2.7. Reflexivity

The study team consisted of qualitative and mixed-methods researchers from multiple racial and ethnic backgrounds. The interviewer (NS) was a Black British doctoral student. Several participants related to NS as a “*helper*” and “*sister*” with inside knowledge of their experiences. This “insider status” may have contributed to the rich data shared, as their visible similarities seemed to have contributed to students feeling comfortable and safe (Tuffour, 2018).

NS found listening to three visibly upset participants’ responses to the SQUIN difficult because she felt compelled to show an emotional response to help them feel safe, despite BNIM guidelines encouraging interviewers to remain neutral during sub-session one (Wengraf, 2001, 2008). One student cried when recalling past sexual harassment from a white classmate but did not want to stop the interview, despite being offered. NS felt she did the student a disservice because she shared words of encouragement. After the interview, NS used critical questions (Supplementary Material 1) and group reflection with the study team to reflect on this interaction. The team concluded that adapting the BNIM technique to actively watching and listening for signs of distress and communicate empathy (e.g., nodding when appropriate) or neutrality (e.g., relaxed facial muscles) was ethically and morally necessary to ensure the participant felt safe, validated and respected.

## 3. Results

**Figure 2.**
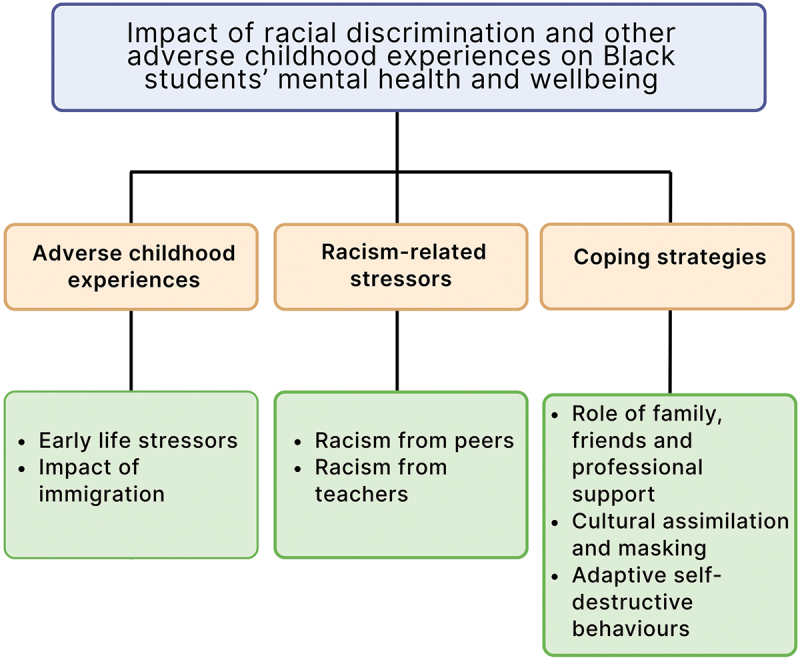
Main themes and sub-themes of pre-university stressors impacting Black students’ mental health and wellbeing.

Three main themes were derived from the data: (i) Adverse childhood experiences, (ii) Racism-related stressors, and (iii) Coping strategies (Figure 2) to answer the research question, what are the life events and experiences that shape Black students’ mental health and wellbeing before commencing UK university studies?

The themes related to the participants’ perceptions of the role adverse experiences, social relationships and discrimination had on their mental health, wellbeing, and educational experiences, and the coping strategies they developed to manage these stressors.

The authors have referred to the participants using their stated preferred pronouns. When referring to a collective experience shared by all or many participants, we have used the gender-neutral terms “their” or “they”.

### 3.1. Adverse childhood experiences

#### 3.1.1. Early life stressors

Participants had been exposed to numerous adverse childhood experiences (ACEs) that caused them physical and psychological harm. Some experienced and witnessed violence, abuse, or neglect by family members, friends or neighbours. Several recalled being separated from or abandoned by a parent or guardian or mourned and grieved the death of a loved one. Many experienced poverty or financial insecurity. A few cared for their parents with serious mental and physical health conditions.

I grew up in a really notorious [housing] estate^2^ for gun violence and drugs. It was really, really deprived and really poor. I had friends that were killed and saw loads of things that I shouldn’t have seen as a child. I don’t think at the time it registered how traumatic it was but I was always really, really scared. I decided I would become a doctor to be able to save my friends who had died or gotten hurt [so] I put a lot of pressure on myself. [In college^3^] I didn’t get the grades [to get into medicine] because I was not in a good space. [because of anxiety] (Mia, Undergraduate)

Mia’s fast speech and repetition of “*really*” mirrored the racing ruminating anxious thoughts she described experiencing due to repeatedly witnessing the murder of loved ones but not having any ability to save them. Mia centred her identity around caregiving and civic duty, perhaps to gain control, meaning, and power in an oppressive environment.

Participants described being perceived and treated as adults by their parents or teachers (i.e., adultification) and being forced to parent their parents or siblings (i.e., parentification). They recalled having very little control over their lives because they were expected to take on adult roles and responsibilities for their family’s survival and wellbeing, leaving little time and energy to be a child. When they reached out to family members or teachers for support for adultification or parentification, they described being invalidated. This resulted in them becoming socially withdrawn, to abscond from school, and to develop anxiety and depression symptoms.

I would try and study really hard, but then I had caring responsibilities with younger siblings, and it was too much [*Ayo huffs*] … At home I’d say “I have to stay in class” and my mum being like “You have these responsibilities” and at school my teachers are like “Why you not paying attention in school?” (Ayo, Doctoral)

Ayo repeatedly sighed and huffed when they recounted the cultural expectation of being expected to parent their six younger siblings and excel in school. Their actions were indicative of their anger and frustration with feeling misunderstood, misrepresented and undervalued. Similarly, all participants described hyper-focusing on their studies as a form of emotional or physical escapism from the stresses of supporting their family, friends or peers.

I was drinking so much coffee, I was waking up at 5:00AM, 4:00 AM to study and revise, do bits and bobs around the house, help [my siblings and mum]. I was in a tunnel vision kind of state, just focused, focused, focused on being the best [student] not just the [house]maid, so I didn’t realise how [distressed] I was until I stopped sleeping, stopped hanging out with my friends, and got really sad all the time. (Naomi, Masters)

Naomi repeated the word “*focused*” and used her hands to create a tunnel, perhaps to emphasize how fixated she was on studying obsessively and achieving good grades as a coping mechanism from stress due to caring responsibilities. For Naomi, and other students, engagement in familial responsibilities and educational self-improvement often came at the expense of childhood joy (i.e., socializing with friends) and wellbeing activities (i.e., good sleep hygiene practices). These sacrifices were informed by their identification with a collectivist culture, within which educational achievements and strong family values were valued, recognized and celebrated by the broader community.

The only thing on my mind was that my people have been through too much and I can’t let them down, I have people depending on me to be the best … the best son, the best student so they can be proud of me. (Sean, Undergraduate)

Despite experiencing many childhood challenges, those who grew up in predominantly Black neighbourhoods described feeling psychologically and physically safe to explore their personal and social identities and seek and utilize community support and resources to manage their wellbeing.

I grew up in a Black area, I went to very Black schools. It was always safe, people weren’t racist, wouldn’t look at you funny, and I got to see the richness of Blackness. You wouldn’t be gaslighted, you wouldn’t get any microaggressions. I didn’t feel like I had to assimilate, I didn’t feel like I had to code switch, I could speak slang or not speak slang. For the first 18 years of my life, I thought that’s how life was and that’s how England was [*Mia pauses and sighs*] until I got to university. (Mia, Undergraduate)

Mia’s description of the predominantly Black childhood neighbourhood and schools sound idyllic. Mia’s sudden change in tone, pitch, and pace when she recollects her discovery that anti-Black racism was societal norm highlights how transitioning from a perceived safe psychological environment to a harmful predominantly white institution can be unsettling.

Another early life stressor a few participants recalled was navigating racialized ableism in school due to having a neurodivergent condition(s). They described feeling anxious and disengaged in classroom and social spaces due to feeling unable to connect with others and feeling victimized for being “different” in terms of race and social norms. All recalled not being diagnosed until later in their education journey, despite struggling in school from an early age. Three participants speculated that their late diagnosis was due to teachers holding racialized stereotypes that Black children are “different” or “weird” and so ignored their neurodivergent symptoms and withheld access to support and resources.

I got my Dyslexia and Autism diagnosis a month before exams and by then it was too late. I was so depressed after that, it knocked me. I am so mad my teachers didn’t do anything about [my neurodivergent symptoms] because when I told my form tutor [about my diagnosis] they said “Oh we knew you were a bit different” and laughed, can you imagine?… I just wish, I wish, someone had helped me. (Kesia, Undergraduate)

Kesia’s anger portrays a deep sense of betrayal and sadness for being neglected and invalidated by her schoolteachers. Her repetition of “I wish” and use of the words “*knocked me*”, which conjured the image of being knocked out after repeated hits in a boxing match, underpinned the cumulative adverse impact that multiple and intersecting forms of discrimination had on her sense of agency and confidence.

#### 3.1.2. Impact of immigration

Participants who immigrated to the UK spoke about feeling lonely, frustrated or upset about the removal of green spaces, sunny weather, community support, and family bonding, all of which had been a source of comfort and joy in their home countries. Most participants experienced their families who had immigrated from Africa or the Caribbean, as “*strict*”, “*angry*”, or physically and emotionally unavailable. They attributed this reality to their families having to prioritize work and repress their stress to survive and provide for them financially in racist and classist conditions.

I was such a happy child in [the Caribbean] and my mum was happy too … When we moved to [the UK] she was so angry all the time, she worked all the time, she would complain about the [white] people she worked with all the time … I hated [white people] too for everything they did to us … I just turned into an angry. [child] (Xavier, Undergraduate)

Xavier stuttered on his words and looked away from the camera, appearing lost in thought. The repetition of “*all the time*” and sadness in his tone indicated his distress following the loss of valuable familial bonding time that they needed for his emotional wellbeing. Similar to Xavier, many recalled feeling unable to express their emotions or psychological distress to their families. Instead, they described being obedient children in an attempt to minimize the stress that living and working in racist environments as immigrants had on their families.

I learned early on that I had to be a good, well-behaved child at all times. I remember one time my teacher threatened to call my mum and I was like, “Please don’t, she’s got enough on her plate, she doesn’t need additional stress from me”. [My mum’s distress] was never verbalised, but you know, you learn to read the room and pick up on emotions. (Amara, Undergraduate)

Amara’s recollection of pleading to her teacher was one example of how she had to learn how to respond to the emotional states of adults in her life for self-preservation because her actions could lead to harsh judgement and punishment. She later described how her continuous state of self-monitoring, restraint and silencing led to her feeling constantly burnt-out, anxious and resentful towards the adults and peers whom she perceived to be perpetuating harm by failing to remove the compounding responsibilities and expectations.

Participants relayed feeling that they had to meet or exceed their family’s expectations of economic and social mobility for their approval, love, and care. Subsequently, they were hyper-focused on their studies and avoided potential distractions to achieve high grades. According to the students, this led to anxiety, fear of failure, or negative self-talk.

I remember talking to [my friend] on the phone and they’re like “I’ll call you back, I have to go eat with my family” [and I said] “Woah, what do you mean eat with your family? Like every evening you guys just sit around the table and eat together?” and they’re like “yeah?” and I am thinking “I use my dining table to study”. In [the Caribbean] we do stuff like that, so that for me was “I wish I had that, but no one has time”. Instead, I spent all my time. [studying] (Umi, Masters)

Umi looked down, shook his head repeatedly, frowned, and was clearly distressed. Perhaps what formerly represented family bonding and cultural connection (i.e., the dining room table) had become merely an object for studying and social and economic advancement. This could be why he was hyper-focused on his studies to fulfil his duty to his family.

Despite these challenges, some participants stated that their transition to university was “*comfortable*” and “*easy*” due to their experiences of immigrating to the UK as children. They believed that this mitigated the feelings of homesickness, lack of confidence, or inability to take care of themselves, which they observed in their peers during the transition to university.

I was really prepared for having to manage my studies and my life by myself, I was really independent. I was really happy to be moving out of the house. (Xola, Masters)

Xola expressed a strong feeling of independence, but their repetition and increased emphasis on the word “*really*” gave the impression that they had to convince themselves and others of their self-efficacy to survive during a stressful transition period.

### 3.2. Racism-related stressors

#### 3.2.1. Racism from peers

Racism from secondary school and college peers disclosed by participants included racial bullying (e.g., name calling or exclusion from playground activities). Participants discussed feeling depressed, anxious or low in confidence and self-esteem at primary and secondary school due to repeated racial bullying about their hair, features, or colour of their skin by white and Black peers. They reported experiencing racial bullying at either predominantly racial/ethnic minoritised or predominantly white schools. Individuals described internalizing the stereotypes, prejudices and discrimination they faced frequently which led to feeling consumed by negative thoughts that they were “*ugly*” and “*worthless*”.

I hated my hair, my nose, my eyes, my body, everything [*Amara shakes her head*]. None of the boys in school thought I was cute, they all ignored me or made fun of me, especially the [white boys], they really thought I was disgusting [*Amara starts crying*] … I would cry to my [older] sister all the time. (Amara, Undergraduate)

Amara stumbled on her words, and her tone became angry but then quickly changed to sadness. Her fluctuation from anger to sadness may be mirroring the powerlessness she felt feeling unable to challenge racism shown by her peers. These complex childhood emotional experiences in response to racial bullying were echoed in many participant’s stories, including those who attended predominantly racial and ethnic minoritised schools.

I would come home from school, and I would feel really, really really, really, really sad, if that makes sense?… I would define it as “OK, I feel a little bit depressed today or I feel depressed this week” or whatever. (Imani, Undergraduate)

Imani repeated “*if that makes sense?*” and “*whatever*” before taking long pauses to look at the researcher throughout her interview. These verbal cues suggested an attempt to minimize her feelings or the need for reassurance or validation. This was made evident when she disclosed being invalidated or gaslit by peers when she attempted to share the detrimental impact colourism had on her mental health.

There was a girl [in my class who] was mixed race and she had lighter hair, she had lighter eyes. To me she was normal, I saw me and her as equal. I noticed in class that none of the guys were talking to me, they’re always flirting with her, they’re always grabbing her hair, grabbing her hand … They spoke nice to her but the little snide comments [they said to me about] how I looked was getting to me. (Imani, Undergraduate)

Imani’s tone became softer and slower, her voice started to shake, and she looked up at the ceiling when she listed the differences in appearance between herself and a light-skinned Black peer. In contrast to the accounts above, some participants believed that attending a predominantly Black school was a protective factor against colourism, featurism and hairism.

Because I grew up around all ethnicities but especially Black people … . I knew I had dark skin, but it was never an issue. No one ever made fun of me because of [my dark skin] at primary school or high school, [my skin colour] was never an issue [because] everyone was dark skinned. (Xavier, Undergraduate)

The difference in Xavier’s account may be because his skin colour was perceived positively by Black and Asian peers, which afforded Xavier likeability and led to a positive self-perception of his Black identity.

#### 3.2.2. Racism from teachers

Participants spoke extensively about their white secondary school teachers treating them poorly or differently from other racially minoritised and/or white peers due to anti-Black racial stereotypes and prejudices. For example, their teachers refused to learn how to pronounce their names correctly or repeatedly mistook Black students for another student of the same race, despite having taught the students for years. Some recalled that their schoolteachers would seemingly purposely hinder their educational development. One individual, who was a prefect in sixth form, recalled that their teacher refused to assign them roles and responsibilities that would enhance their university application.

You start to question, “Am I stupid? Am I dumb? Is that why they’re not giving me work? Do they not think I’m capable of doing things?” I spoke to the Indian girls who were [prefects] with me and they didn’t understand what I was saying, one actually reported me to the headteacher and I was given detention for being rude. [My Indian peers] were always treated so much better because they were quiet and always got good grades and made the school look good. I started to hate them, I hated everyone, I hated myself … it hurt. (Amara, Undergraduate)

Amara’s story echoes the “model minority myth” stereotype which posits that specific minority groups are superior in educational, economic and social outcomes. Amara hit her chest with a clenched fist when she said: “*it hurt*”. This action seemed to depict that the teacher encouraging competition for access to support and resources (i.e., educational enrichment activities) between peers from different racial minoritised identities was similar to a weapon used to harm her.

Many recalled that some schoolteachers acted on the stereotype that Black students are not intelligent, as evidenced by the teachers marking the participants’ work more critically than their white peers or accusing them of cheating when they achieved a high mark on their assignments. These teachers were said to have instilled fear and anxiety in the participants because, without favourable grades, they would have found it difficult to progress through education.

I had a full certificate with Bs and then my teacher gave me an F and he said to me ‘you should go and do an apprenticeship as a hairdresser because you don’t have the intellect to do A-levels and literally, he said, “your parents don’t read Dostoyevsky with you” … I think that’s when my mental health story started. I really felt like, I’m not worthy, I’m a second-class citizen, I don’t belong here, my teachers don’t believe in me. (Clara, Doctoral)

Clara became sentient as a child that white teachers held a position of authority and power over her because they could dictate her grades, regardless of how she performed in the classroom or her assignments. The teacher’s choice of Dostoyevsky to shame and mentally harm Clara is ironic, considering the author’s famous depiction and lived experiences of mental illness. While recalling this memory she laughed, shook her head, and threw her hands in the air as if to imitate the powerlessness and frustration she felt.

Similarly to Clara, participants recalled feeling powerless to challenge their teachers’ racist behaviours. Participants attempted to disprove their teachers’ negative prejudices about their ability to achieve high grades or attend university by hyper-focusing on their studies, which led to maladaptive perfectionism. Subsequently, their identities and self-worth became associated with their achievements and contributions to educational institutions (e.g., winning prizes or excelling in multiple student committees and roles).

My teachers were useless, they didn’t think I would amount to anything, but I didn’t want to let myself down, my family down, my friends down, because everyone had really invested in me … The pressure got to me, and I was burnt out before I even got to university. (Xavier, Undergraduate)

Xavier’s use of the words “*amount to anything*” and description of himself as an investment seemingly evidenced his childhood belief that educational success would give him the social and economic value, worth and belonging he needed from adults. Xavier’s account was also echoed in Malik, Sean, Umi, and Tajo’s stories. They all described being impacted by racialized toxic masculinity. They described living with a daily threat of being the perpetrator, witness or victim of violence or social rejection at school, which left them unable to express their emotions to their peers due to fear of bullying. In his story, Sean repeatedly used the term “toxic masculinity” to refer to emotionally, financially, and socially damaging expectations and norms placed on him as a young Black boy.

I feel like as a Black boy, there’s a lot of pressure to succeed and to prove people wrong. I had teachers who thought that I would be in prison by the age of 18, or that I couldn’t amount to much. [Toxic masculinity] has a really negative psychological effect because as a teenager [toxic masculinity] makes you want to succeed, getting involved in illegal activities just to show you have money and you’re doing well and successful to compensate for the fact that you don’t feel good enough. (Sean, Undergraduate)

As a young boy, Sean believed that money would bring social privileges and help him transcend racial stereotypes. Whilst recalling his story, his voice became deeper, his speech slowed, he shook his head repeatedly and looked down. His presentation suggested that racialized toxic masculinity left him feeling vulnerable, depressed, and alone.

### 3.3. Seeking support and coping strategies

#### 3.3.1. Role of family, friends and professional support

Most participants expressed disappointment that their parents could not provide them with the advocacy and emotional support they needed for the racism they had suffered at school. Many described multi-generational transfer of trauma from their grandparents to their parents, as the reason for not being offered appropriate support.

My parents were really fearful of the repercussions [of reporting racial discrimination]. “No, you can’t sue”, “No, you can’t [report racism from white people with power and authority]”, “we can’t get into arguments [with white people]”, “what if they’re going to [physically harm] us? What if they’re going to take our citizenship away?” my parents were really fearful, so [reporting racism] never happened, but my motivation to perform in school just went downhill. (Clara, Doctoral)

Here, Clara’s voice became angry, and she huffed repeatedly when she recalled her immigrant parents’ fear of punishment from white authority if they swayed from the model minority trope (i.e., silent and obedient when faced with oppression). Her anger quickly turned to sadness when she recalled becoming disengaged from education. Her reactions seemed to illustrate her feelings of powerlessness against white authority figures as a child, and simultaneous resentment towards her parents who could not protect her because they also felt powerless. Importantly, participants who described having a white parent who taught them how to navigate racist education systems said this knowledge did not protect them from observing or experiencing racism, classism, or ableism.

Kipo was the only participant who spoke about receiving therapy before attending university, which she had accessed through the private school she attended. However, her experiences of racism from white family members, teachers, and peers left her feeling unsafe and uncomfortable talking to white mental health professionals about her racial trauma, which left her unsupported.

I went to therapy [for my eating disorder] and pretended to be cooperative, but [I did] not actually cooperate. I was sitting in front of white English Psychiatrists who I recognised very early on were not going to understand me [a Black girl] or my issues caused by racism … .As soon as I then got [referred to] therapy I was like “my parents can’t afford this so what I need to do is just get to a good enough weight where they will leave me alone”. (Kipo, Doctoral)

Kipo’s use of the words “*leave me alone*” and stern tone of voice demonstrated her fear and mistrust of adults, which was echoed in the other participants’ stories. She shunned engaging in treatment to protect herself from further psychological harm and instead suffered in silence.

Most participants recalled the benefit of having Black friends or family members who provided spaces where they could receive respite from racism or abuse. These places were outside of school spaces, at their friends’ family houses, during team sports, or in green spaces such as parks.

[Black students] would be disciplined by teachers way more than anyone else would [for example] the times I had a Black peer in my class they would separate us to keep us feeling alone … What helped is I had a really, really small tight group of friends and a brother and we helped each other [study] and stay the course. [to university] (Aba, Doctoral)

Aba being singled out by high-school teachers reinforced racial stereotypes and prejudices that Black students cannot be trusted to behave according to preferred white cultural norms in learning spaces and should therefore be silenced and remain silent. This might explain why Aba later described feeling unable to access community-building opportunities in learning spaces at college and instead relied on Black peers and siblings to act as psychological buffers against internalizing inferiority messages from white teachers with assigned authority.

#### 3.3.2. Cultural assimilation and masking

Participants mentioned that they learned how to cope with their psychological distress during secondary education by censoring their thoughts, feelings, and behaviours (i.e., masking).

I detached myself, I was quite young, but I told myself “you don’t cry about this anymore, [my dad] is the way he is”. It was a repeated rollercoaster of me being disappointed and upset and then just numb, and I went through that cycle quite a lot. (Ayo, Doctoral)

Ayo described constant overwhelming feelings of psychological distress growing up with an abusive father. Her use of the words “*roller coaster*” is a vivid metaphor to convey that her cycle of disappointment, upset, and numbness was disorientating and destabilizing, which imitated the twists and turns of a roller coaster. Using the word “*repeated*” to describe the “*roller coaster*” depicted her feelings of helplessness and lack of control over the recurring abuse. Ayo’s process of “*mentally detaching*” herself from psychological distress was echoed in the other participants’ stories. Some participants seemed to cope with relentless daily racism at school by minimizing the ill intentions of others.

Racial microaggressions or when people asked [my Black peers] certain questions, or touch our hair, it’s more understandable when you’re younger. People were fascinated by [our hair]. That’s kind of how we, [my Black siblings and friends], looked at [racial microaggressions] when we were younger, we try not to let those type of things bother us like mentally, but it did make us anxious. (Aba, Doctoral)

Aba’s normalization of microaggressions indicated an attempt to minimize the repeated invasive behaviours performed by adults towards Black children to manage his discomfort and distress. However, as Aba later mentioned in his accounts, masking and avoidance were not optimal for practising socializing skills and building social networks.

I find it very difficult to open up to others and say “Yo, I’m struggling, what about you? Are you also struggling? Because at the moment I’m finding it difficult” … . I was bottling up everything, all emotions, all difficulties. From young I recognised people’s reactions when I’m smiling, they think “Zuri is fine”, so I smiled. That’s what I continued doing, smile, smile, smile, smile your way through it. I thought my emotions are too messy and too ugly for me to share, so let me just put on my smile because that’s not ugly, that’s not messy … I ended up very lonely and depressed. (Zuri, Masters)

After expressing her difficulty asking for support for distress, Zuri’s casual expression (i.e., “*yo*” and simplicity of the question), when saying what she wished she could say suggested low agency and learned helplessness. Her repetition of “*smile*” after she disclosed going through a period of difficulty may be interpreted as a mantra to remain steadfast in her coping strategy. Her choice to use “*messy*” and “*ugly*” to describe her emotions may depict her internalization of learned familial and cultural beliefs about psychological distress.

Others described “*assimilating to survive*” compulsory education. Examples of cultural assimilation included changing their accent, dialect, and body language; playing popular British playground games; and remaining silent rather than participating in classroom activities.

I had to get very good at assimilating into spaces because I was the only Black person, and I was subject to racism. I had to learn to have quick comebacks to shit people said and to hold my own … one thing I was always good at was socially fitting in or playing the game or assimilating into whatever I needed to do to survive that experience, whereas my actual feeling was I actually don’t want to be friends, I don’t want to be. [at school] (Kipo, Doctoral)

The narrative amongst participants, as illustrated in Kipo’s story, was that assimilation was survival in predominantly white spaces. Not adapting came with the risk of being bullied by peers and punished by teachers and their families. However, assimilation left participants feeling resentful towards their white peers. Contrastingly, Leah described refusing to assimilate as a child because her family taught her that maintaining her cultural and racial identities was a form of resistance against racism.

[My grandmother would say] Don’t let [white people] mess you around, don’t take no for an answer, if you don’t ask you don’t get, if you don’t work hard you don’t get anything, you better work hard, I don’t want excuses … and don’t rely on anyone… My nan said, “I’ve had to fight to get [to England] and you’re going to fight as well”. (Leah, Doctoral)

Similarly to Leah, many participants said they adopted hyper-independence to cope with poor mental health during school after people in authority (i.e., teachers or parents) repeatedly failed to meet their physical or mental needs. Others were fearful of disappointing their families to whom they had parental responsibilities (e.g., younger siblings) or feared embarrassment or rejection from their peers.

#### 3.3.3. Adaptive self-destructive behaviours

This theme discusses the paradox of seeking autonomy and emotional healing through self-destructive actions. Many participants hyper-focused on their studies to physically escape toxic spaces at home and in school, and to avoid thinking about their mental distress. For many, their self-esteem and self-worth was attached to education because performing well in school elicited love and care from their family or teachers. According to them, they consequently developed an association between positive feelings about themselves and striving and thriving in their studies.

Education was an outlet to me, I go to school, I do my work, I was thriving. I got to high school, they bumped me up to all the like top classes, top set … .I’ve always been quite academic … my dad, he ingrained in me a strong sense of the importance of education, so I was always like, ‘I have to be someone’… and you know being a Black woman in this world, you have to push. (Ayo, Doctoral)

Eventually, participants described feeling overwhelming pressure to continue excelling in their school studies and to disprove the racial stereotypes teachers held against them, which manifested into health risk behaviours such as eating disorders, skin conditions, and substance misuse.

I was drinking an unnecessary amount of [alcohol] with my friends after school before I even got to university. No one [in my family] knows about the stuff [drinking and sex] that I got up to at school, it would kill them [*Naomi pauses*] it nearly killed me … .[Alcohol and sex] was the only way I knew how to make the [anxious] thoughts go away. (Naomi, Masters)

Naomi used alcohol, sexual activities, and restrictive eating to regulate her feelings of helplessness, anxiety, anger, and sadness. However, Naomi later described that these health risk behaviours led to poor nutrition and vitamin deficiencies, which left her fatigued and maintained her stress and anxiety. Others described coping with their feelings of powerlessness by controlling their food, which, for some, developed into eating disorder symptoms.

If you don’t have a strong mother figure who is absolutely secure in who they are [*pause*] it’s tricky [to not dislike your body]. My Mom was always saying she was ugly, but she is very pretty and very slim. So, I said “well if you are ugly, what does that make me?” I didn’t understand, I didn’t get it. (Leah, Doctoral)

Leah’s exposure to constant negative body image narratives appeared to be grounded in internalized misogynistic tropes (i.e., “*pretty*” and “*slim*” are associated with beauty and value). To conform to culturally normative beauty ideals, Leah self-managed by restricting her eating. Later, Leah described struggling with how they perceive themselves (i.e., “*a beautiful curvy Black girl*”) compared to how her mother and peers perceive them (i.e., “*ugly*”) and restrictive eating and worrying about flaws in their appearance may have been a way to manage this cognitive dissonance.

Many participants recalled that hyper-focusing on their studies left them exhausted and over time developed into performance anxiety, maladaptive perfectionism (i.e., setting unrealistic standards), and depression symptoms. Over time, some participants described difficulty engaging with their studies (i.e., focusing during class or completing assignments and examinations), which impacted their grades. Eventually, they recalled absconding from school because studying at home or in a community space (e.g., a library or café) felt safer and more comfortable than being a minority in a predominantly white and racist school environment.

In year 10 my grades got worse, when I got to year 11, in the first term I skipped one third of the classes. I just didn’t go because I felt [*Clara shouts*] “What am I doing here?” I get a B in an exam and the teacher gives me an F and says my “participation in class was a U so we can justify an F” [and] you can’t do anything about it because the teacher has the ultimate power. (Clara, Doctoral)

Clara reflected on the power imbalance and lack of agency she had against white teachers who misused and exploited their assigned power to adversely impact her educational experiences and grades, both of which were known to teachers to have an important influence on Clara’s academic future. Her raised voice and rhetorical questions signify a learned helplessness response, questioning her value, worth, and capability in an institutionally racist environment.

Some students attempted to regain control of their lives by not starting university immediately after compulsory education ended, because they became disillusioned by education. Their decision to evade higher education to avoid facing similar race-based stress was framed as a coping strategy to heal from the psychological distress caused by toxic educational environments.

I was terrified of going to university because I thought if it’s anything like [college], it’s not worth the money [and] the stress. I decided to work instead, and I loved it because everyone just got on with their job … [staff] treated me with respect. (Sean, Undergraduate)

The use of “*terrified*” signified how profound his fear of anticipated continual racial trauma was that it outweighed his goal of studying his dream course at university. Sean’s search for respect may reflect his desire for acceptance, to feel valued and not judged by stereotypes to enhance his self-worth and reduce the distress he recalled during school.

A few participants described having to work immediately after school to contribute to household bills and because they did not have enough money to pay for the university course fees. They reported eventually starting university for social and economic mobility to “*escape the cycle of childhood poverty* (Xola)”.

I found it difficult to get off benefits, working and with a child. That’s not a race thing, but benefits [universal credit and jobseeker’s allowance] are like a noose around your neck. Anyone on benefits in this country, it’s very hard to get off [benefits], it’s virtually impossible. [Years later] the only reason I trained to be a teacher [was] so I wouldn’t have to [financial] worry about my daughter. (Leah, Doctoral)

The vivid metaphor of UK welfare benefits being a “*noose around your neck*” emphasized how much being at the intersection of racism and classism made her feel suffocated, powerless, and doomed to a life of adversity.

## 4. Discussion

The current study highlighted how Black students’ experiences of racial discrimination and other adverse childhood experiences (ACEs) impact their mental wellbeing in education. The study also explored how Black students attempt to survive and thrive in education despite these pervasive experiences. Interestingly, there was a meaningful consensus among participants, despite differing ages and level of education, possibly because racial inequalities and discrimination have been persistent in UK education institutions since the 1940s post-war period of post-colonial migration and settlement (Gillborn et al., 2016). The only relevant difference was that older participants were observed to be more comfortable speaking about personal experiences of poor mental health in sub-session two, after prompts from the interviewer possibly due to generational differences in perceptions and language use around psychological distress (Clarkin et al., 2024).

In accordance with the Culturally-Informed Adverse Childhood Experiences Framework (C-ACE) (Bernard et al., 2020), the study findings highlight how historical racism (e.g., generational trauma and cultural stigma) may result in Black students growing up in potentially stressful environments (e.g., several participants reported living in poverty, immigration stress, and/or witnessing violence) with an increased risk of biopsychological vulnerability (e.g., hyper-vigilance to *anticipated* racism). These exposures can increase Black students’ risk of ACEs (e.g., many experienced abuse, neglect and racial discrimination), which, in turn, exposes them to racism-informed conditions (e.g., lack of control over educational attainment due to their teacher’s racial stereotypes), exacerbate biopsychological vulnerability (e.g., hyper-vigilance to *expected* racism), and health risk behaviours (e.g., some participants developed disordered eating, substance misuse, or absconded from school). These cumulative exposures resulted in negative mental health outcomes (e.g., depression and/or anxiety) and increased vulnerability to future ACEs from early education through to university. Notably, emotional dysregulation and poor school educational attainment limited some students’ access to further education and work opportunities, exacerbating their psychological distress and maintaining social and economic subjugation. The findings add to the literature by indicating that Black students may report health risk behaviours (e.g., poor sleep hygiene, alcohol misuse, and/or skin-picking) and avoidant behaviours (e.g., truanting or disengaging from classroom activities) to self-soothe and protect Black students from future ACEs by motivating them to avoid expected harmful people, spaces, and feelings, allowing them to succeed in education.

Black students described feelings at the mercy of powerful and privileged white teachers who held decision-making power over students’ grades and their future educational and work prospects. Concurrent to Ray’s “Theory of Racialised Organizations” (Ray, 2019), the current study findings suggest that (i) racialized UK schools diminish Black students’ feelings of control over their actions and the consequences of their actions; (ii) the toxic and hostile school cultural norms cause and maintain the unequal access and distribution of resources and support; (iii) whiteness comes with automatically assigned power and privileges to resources and support; and (iv) school teaching staff routinely break policies and procedures that are meant to protect Black students from discrimination because the policies and procedures are loosely enforced.

In an attempt to regain control and power from abusive adults or peers, participants talked about employing the following coping mechanisms. They became hyper-organized, hyper-independent, studied excessively, engaged in restrictive eating behaviours, or absconded and/or disengaged from school. According to qualitative studies, young people are likely to endorse individualistic coping strategies because of mistrust towards adults, concerns about being judged, and low mental health literacy (Pimenta et al., 2024; Stapley et al., 2023). Longitudinal studies in the US have also documented that repeated exposure to ACEs may cause students to compromise their sleep, exercise, and other aspects of self-care, which can result in physical health problems, including obesity and high blood pressure (Brody et al., 2016, 2018; Chae et al., 2020; Hatch et al., 2011; Heard-Garris et al., 2024). The study findings emphasized that Black students may report health risk behaviours (e.g., poor sleep hygiene, alcohol misuse, skin-picking) to self-soothe and as a consequence of hyper-focusing on their studies to disprove their teacher’s negative racial stereotypes and obtain access to vital resources (e.g., fair assessment). Black children navigating the UK educational system where they have minimal assigned power or control might explain why Black students are more likely to progress to university than average, but less likely to obtain high grades, enter Russell Group universities, and progress to postgraduate education (Roberts & Bolton, 2023).

### 4.1. Impact and implications

The findings of this current study add value to existing research by revealing how the psychological and emotional energy required for Black UK children to manage racism, misogynoir, toxic masculinity, and ableism in school can lead to poor mental wellbeing before students transition to university.

Schools might consider offering Black students culturally informed childhood trauma assessments and Trauma-Focused Cognitive Behavioural Therapy (TF-CBT) for individuals, groups and families (Metzger et al., 2020). Culturally-informed TF-CBT can help Black children to identify feelings associated with experienced and anticipated racial discrimination, provide strategies to manage distress, consider culturally relevant forms of trauma processing and narration, and create a safety plan for future experiences of racial discrimination (Lee et al., 2024; Metzger et al., 2020). Offering students the choice of race-matching appears essential for autonomy and therapeutic alliance given the study finding that experiences of racism might leave Black students feeling uncomfortable and unsafe to receive therapeutic support from a white mental health professional. Professional bodies for psychology need to ensure that training, pedagogy and curricula operate from an anti-racist framework. The accounts provided in this study indicate that silencing and reducing the severity of racism on school children can be traumatic, and re-trigger earlier experiences of racial oppression, further compounding the mental distress encountered. Although mental health practitioners might have supported Black students with race-related trauma, very few would have been taught how to assess, talk about, or treat racial trauma.

Schools could build African Caribbean Societies (ACS) for Black students to discuss historical and current cultural and racial topics that impact their mental health as racial socialization has been found to reduce distress and contribute to academic adjustment and performance among Black students in the US (Anderson, Jones, et al., 2019; Anderson, McKenny, et al., 2019; Golden et al., 2021; White-Johnson, 2015). Funded ACS groups that are designed and run by Black student champions might be beneficial for students such as those interviewed for this study who seek social support and guidance from culturally safe and familiar peers.

**Figure 3.**
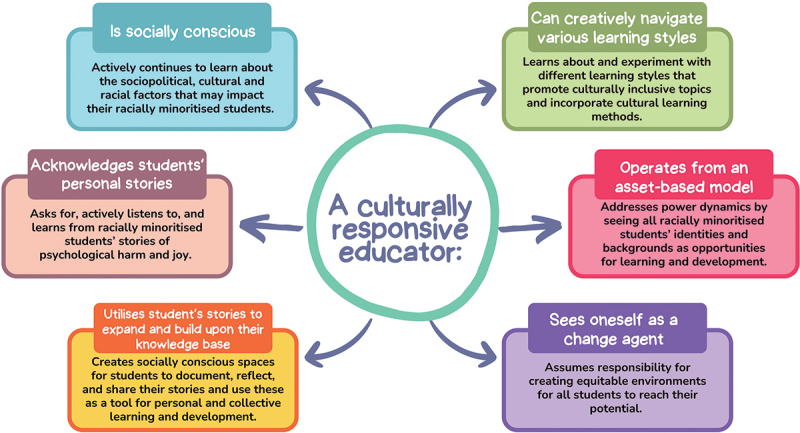
A summary of the six aspects of a culturally responsive educator by Villegas and Lucas (2002) with specific traits of a racially and culturally responsive educator based on the study findings.

Finally, schoolteachers may benefit from training and resources to reflect on their assumptions and biases to become culturally responsive educators (Figure 3). By recognizing and challenging the inherit power dynamics in their roles and their own social and cultural identities, staff can seek to minimize Black student’s feelings of othering, unbelonging, distress and powerlessness in education. This might enhance Black students’ ability to continue with and engage in educational spaces and improve their mental wellbeing.

### 4.2. Limitations and future directions

The sample was self-selecting, so the study may have captured individuals with particularly strong views about racial inequality in student mental health and arguably cannot know how representative their views are of the wider Black student population. However, neither “generalisability” nor reliability is the driving force of IPA research because the belief that findings can be replicated in different contexts, to different people, at different times is counter-intuitive to most qualitative research (Willig, 2014). While the narratives of older participants may be subject to recall bias (Colombo et al., 2020), BNIM interviewing prioritizes the exploration of how individuals make sense and narrate their recollected experiences, rather than the factual accuracy of those memories.

Future research is needed to explore how white and Asian students and staff view their own and others’ racial and cultural identities in relation to mental health experiences in education. This might provide insight into how to develop a racial trauma awareness course for students and staff to improve inclusion, belonging, and integration. Researchers might consider using a similar adapted BNIM interview method because offering a space for Black students to tell their story uninterrupted, in the way they felt comfortable, was an important part of the research process, especially as such few spaces existed for Black UK students to express vulnerability (Stoll, Jieman, et al., 2023). Furthermore, survey data could improve understanding of whether and how mental wellbeing experiences differ across different Black student identities (including gender, culture, ethnicity, and nationality). This is important given the study finding that Black students’ mental health experiences within institutionally racist education systems are not heterogeneous because of varying cultural experiences. In addition, having a better understanding of the stressors that Black students’ families face and how they overcome these challenges can help improve the information, support, and resources provided to family support systems. A longitudinal mixed-methods study utilizing national data on Black student cohorts could help examine how exposure to racial discrimination and other adverse experiences impacts mental and physical health outcomes from nursery school through to graduation.

## 5. Conclusions

The study aimed to explore the life events and experiences that shape Black students’ mental health and wellbeing before commencing university studies. This study adds to the international literature by revealing how racism, classism, sexism, and ableism within UK schools persist at the expense of Black students, who encounter these oppressions through compounding racism-based stressors and other adverse childhood experiences. These repeated exposures can influence and be influenced by poor mental health, and the coping strategies they adopt to survive and thrive in UK education. The study builds on the “Culturally-Informed Adverse Childhood Experiences Framework” to show why Black school students might engage in health risk behaviours to cope with UK-specific institutional systems that perpetuate inequalities. Institutions can invest in self-reflection tools, funding, and resources for culturally appropriate interventions and enhance professional psychology courses to be anti-racist.

## Supplementary Material

Supplementary Material 1_anonymous_QHW.docx

## Data Availability

The participants of this study did not give written consent for their raw data to be shared publicly, so due to the sensitive nature of the research supporting data is not available.

## Notes

1. A Pupil Referral Unit (PRU) is an alternative education setting in the UK designed to support children and young people who are struggling to engage in mainstream school, often due to behavioural, emotional, or medical needs.

2. A housing estate is a planned group of homes and other buildings built together as a single development, sometimes by local governments to provide affordable housing.

3. In the UK, “college” refers to a further education institution, often a sixth-form college providing A-level or equivalent courses required to attend university. A college does not refer to a university, as it often does in the US.
