# Supplementary material for: Impact of racial discrimination in education and other adverse childhood experiences on black students’ mental health and wellbeing: an interpretative phenomenological analysis study
Source: Int J Qual Stud Health Well-being. 2025 May 23;20(1):2507754. doi: 10.1080/17482631.2025.2507754 (PMC12107663; doi:10.1080/17482631.2025.2507754)
Supplement: Supplementary Material 1_anonymous_QHW.docx [file ZQHW_A_2507754_SM7951.docx]

SUPPLEMETARY MATERIAL 1: Reflexive questions for conducting qualitative health research, created by the Black Student Wellbeing Study research team.

| **Reflexive questions for conducting qualitative health research, created by the Black Student Wellbeing Study research team.** |
| --- |
| 1. What does my personal story tell me about why I want to research on Black students’ mental health? 2. What have I learned from my experiences and observations that might facilitate the research process? 3. What role do the study design and analysis methods play in constructing the data and findings? 4. How has the current sociopolitical context shaped the research process? 5. Would investigating the research questions differently have given rise to a different understanding of Black students’ mental health? 6. What might it be like for a Black student to talk to me, a visibly Black female student, about their mental health experiences? Depending on their visible identities (e.g. gender, race, ability, colour, nationality, accent etc.) 7. What types of personalities and stories am I drawn to, and which do I find harder to listen to and engage with? 8. How might my assumptions about power and powerlessness impact the relationship between myself and the participant? 9. What themes do I assume will come out of the interview? 10. How will I be able to tell in the moment that my personal experiences are impacting my research process (e.g. interview, data interpretation, choosing quotes)? |
